# Ablation of the Cardiac-Specific Gene Leucine-Rich Repeat Containing 10 (*Lrrc10*) Results in Dilated Cardiomyopathy

**DOI:** 10.1371/journal.pone.0051621

**Published:** 2012-12-07

**Authors:** Matthew J. Brody, Timothy A. Hacker, Jitandrakumar R. Patel, Li Feng, Junichi Sadoshima, Sergei G. Tevosian, Ravi C. Balijepalli, Richard L. Moss, Youngsook Lee

**Affiliations:** 1 Department of Cell and Regenerative Biology, University of Wisconsin-Madison, Madison, Wisconsin, United States of America; 2 Molecular and Environmental Toxicology Center, University of Wisconsin-Madison, Madison, Wisconsin, United States of America; 3 Department of Medicine, University of Wisconsin-Madison, Madison, Wisconsin, United States of America; 4 Department of Cell Biology and Molecular Medicine, University of Medicine and Dentistry of New Jersey, Newark, New Jersey, United States of America; 5 Department of Physiological Sciences, University of Florida, Gainesville, Florida, United States of America; University of Western Ontario, Canada

## Abstract

Leucine-rich repeat containing 10 (LRRC10) is a cardiac-specific protein exclusively expressed in embryonic and adult cardiomyocytes. However, the role of LRRC10 in mammalian cardiac physiology remains unknown. To determine if LRRC10 is critical for cardiac function, *Lrrc10*-null (*Lrrc10^−/−^*) mice were analyzed. *Lrrc10^−^* ^/*−*^ mice exhibit prenatal systolic dysfunction and dilated cardiomyopathy in postnatal life. Importantly, *Lrrc10^−/−^* mice have diminished cardiac performance in utero, prior to ventricular dilation observed in young adults. We demonstrate that LRRC10 endogenously interacts with α-actinin and α-actin in the heart and all actin isoforms in vitro. Gene expression profiling of embryonic *Lrrc10^−/−^* hearts identified pathways and transcripts involved in regulation of the actin cytoskeleton to be significantly upregulated, implicating dysregulation of the actin cytoskeleton as an early defective molecular signal in the absence of LRRC10. In contrast, microarray analyses of adult *Lrrc10^−/−^* hearts identified upregulation of oxidative phosphorylation and cardiac muscle contraction pathways during the progression of dilated cardiomyopathy. Analyses of hypertrophic signal transduction pathways indicate increased active forms of Akt and PKCε in adult *Lrrc10^−/−^* hearts. Taken together, our data demonstrate that LRRC10 is essential for proper mammalian cardiac function. We identify *Lrrc10* as a novel dilated cardiomyopathy candidate gene and the *Lrrc10^−/−^* mouse model as a unique system to investigate pediatric cardiomyopathy.

## Introduction

Heart disease is the leading cause of morbidity and mortality in the developed world [1]. However, the molecular events that govern normal cardiac function and the pathological signals that mediate heart disease and heart failure remain largely unknown. The most common form of cardiomyopathy is inherited or acquired dilated cardiomyopathy (DCM), which is defined by ventricular dilation and systolic dysfunction, and is associated with an increased risk of sudden death [2]. While the genetic causes of hypertrophic cardiomyopathy are predominately mutations in sarcomeric proteins, the molecular etiology of DCM has been linked to a wider range of genes including sarcolemmal and nuclear envelope genes and a growing number of Z-disc and cytoskeletal genes [2], [3]. Therefore, determination of the genetic causes of DCM will enhance the understanding of molecular mechanisms leading to pathogenic remodeling of the heart and the development of new therapeutic strategies to treat heart disease.

Leucine rich repeat containing 10 (LRRC10) was identified as a cardiac-specific factor in mice, zebrafish and humans [4], [5], [6], [7] that is robustly expressed in the developing and adult heart [4], [7]. Although Lrrc10 plays critical roles in cardiac development and function in zebrafish [6], the function of LRRC10 in mammalian hearts remains to be elucidated. LRRC10 belongs to a diverse superfamily of leucine rich repeat containing proteins (LRRCs), which contain multiple LRR motifs that form solenoid-shaped structures ideal for protein-protein interactions [8]. LRRCs have been implicated in a wide range of cellular functions, including signal transduction, cell adhesion, DNA repair, development [8], ion channel regulation [9], and mechanical-stretch sensing [10]. LRRC10 lacks any known functional motifs other than its seven LRRs, representing a unique member of the LRRCs.

LRRC10 exhibits a striated expression pattern that colocalizes with Z-disc and sarcoplasmic reticulum markers in adult cardiomyocytes by immunostaining [4]. Electron micrographs show that LRRC10 localizes predominantly to the diad region where the sarcoplasmic reticulum interacts with the transverse tubule, adjacent to the Z-disc [4]. The Z-disc is the protein-rich lateral boundary of the sarcomere where actin myofilaments are crosslinked by α-actinin [11]. Thus, the Z-disc is not only responsible for lateral force transmission between sarcomeres, but also provides a mechanical link from the Z-disc myofilament to proteins in the peripheral subsarcolemmal costamere and eventually sarcolemma and extracellular matrix [3], [12]. In addition to the structural role imparted by the Z-disc, the Z-disc plays a critical role in sensing and transducing signals in response to biomechanical stress in the cardiomyocyte [3], [13]. Genetic ablation of several Z-disc and costameric proteins results in DCM in mice, including deletion of Cypher/ZASP [14], muscle LIM protein (MLP) [15], enigma homologue protein (ENH) [16], integrin-linked kinase (ILK) [17], or vinculin [18]. Further, mutations in Cypher/Zasp [19], MLP [20], nexilin [21], myopalladin [22], ILK [23], and desmin [24] have been found in human DCM patients, suggesting a prominent role for dysfunction of Z-disc and costamere proteins in the pathogenesis of DCM.

We hypothesized that LRRC10 is essential for mammalian cardiac function and tested this by analyzing the basal cardiac phenotype of *Lrrc10^−/−^* mice. Here, we demonstrate that genetic ablation of *Lrrc10* in mice [25] results in prenatal cardiac dysfunction and the development of DCM in early postnatal life. We show that LRRC10 physically interacts with actin isoforms and α-actinin, implicating LRRC10 as a biomechanical link between the myofilament Z-disc and actin cytoskeleton. Thus, work presented here identifies the cardiac-specific factor *Lrrc10* as a novel DCM candidate gene and the *Lrrc10^−/−^* mouse model will provide a unique system to investigate molecular pathways leading to early onset DCM.

## Experimental Procedures

### Animals and Echocardiography

Generation of *Lrrc10^−^* ^/*−*^ mice and genotyping by PCR were described previously [25]. Mice used in this study were generated by backcrossing *Lrrc10* ^+/*−*^ mice with pure C57BL/6 wild type (WT) mice at least six times as described [25]. All experiments employed *Lrrc10^−^* ^/*−*^ mice and littermate or age-matched WT controls maintained on the same C57BL/6 genetic background. All procedures were performed in accordance with the Guide for the Care and Use of Laboratory Animals (NIH) and the University of Wisconsin Research Animal Resource Center policies. Procedures were approved by a University of Wisconsin-Madison Institutional Animal Care and Use Committee (Protocol #M01461).

Transthoracic echocardiography was performed on mice under 1% isofluorane gas anesthesia using a Visual Sonics 770 ultrasonograph with a 30 or 40-MHz transducer (RMV 707B) (Visual Sonics, Toronto) as described previously [26]. Two-dimensionally guided M-mode images of the left ventricle (LV) and Doppler studies were acquired at the tip of the papillary muscles. LV mass-to-body weight ratio (LV/BW), LV dimension in diastole (LVIDd), thickness of the posterior walls in diastole, and isovolumic relaxation time were recorded. Endocardial fractional shortening was calculated as (LVIDd-LVIDs)/LVIDd ×100, where LVIDs is LV dimension in systole. All parameters were measured over at least three consecutive cycles. To evaluate embryonic cardiac function, M-mode images were obtained transcutaneously from anesthetized pregnant females in utero on embryonic day 17.5 (E 17.5) as described [27].

Mice were euthanized by cervical dislocation to isolate hearts for biochemical analyses. Cardiomyocytes were isolated by retrograde heart perfusion as described [28].

### Yeast Two-Hybrid Screening

Yeast two-hybrid screening was performed using full-length mouse LRRC10 as bait at the Molecular Interaction Facility at the University of Wisconsin-Madison Biotechnology Center as previously described [29]. cDNA libraries screened were human heart and mouse embryo (Clontech). Recovered plasmids from growing colonies were subjected to confirmation mating experiments and cDNAs were determined by BLASTing against NCBI Genbank. 14 positive clones were identified as putative cofactors of LRRC10.

### Histology and Immunohistochemistry

Hematoxylin and Eosin (H&E) staining was done as described previously [6]. Masson's trichrome (Sigma) and TUNEL (Millipore) staining of cardiac sections were performed to detect collagen deposition and apoptosis, respectively, according to the manufacturer's instructions. For cell proliferation assays, immunostaining of cardiac sections was performed by incubating with anti-phosphohistone H3 (p-H3) (1∶1000, Upstate, 06–570) and anti-α-sarcomeric actin (1∶100, Sigma, clone 5C5) antibodies as described previously [30]. For coimmunostaining experiments, adult mouse ventricular cardiomyocytes were isolated as described previously [4], fixed in methanol, and incubated with anti-α-sarcomeric actin (1∶1000, Sigma) and anti-LRRC10 (1∶200) [4]. The LRRC10 antibody was previously characterized [4]. Primary antibodies were detected with Alexa Fluor secondary antibodies (1∶4000, Invitrogen) and Hoechst 33342 (0.2 μg/mL, Invitrogen) was used as a nuclear stain.

Cardiomyocyte cross-sectional area (CSA) was measured in wheat germ agglutinin (WGA) (conjugated to Oregon Green 488, 10 μg/mL, Invitrogen) stained cardiac sections costained with phalloidin (conjugated to Alexa fluor 546, 165 nM, Invitrogen). CSA was evaluated in at least 400 cardiomyocytes per animal from identical areas of the middle of the left ventricular wall. Cardiomyocyte length and width were quantified in at least 100 isolated ventricular cardiomyocytes per animal. Images of heart sections and isolated cardiomyocytes were taken with a Zeiss Axiovert 200 microscope and Zeiss AxioCam and morphometric quantitiation was performed using NIH Image J. Images of immunostained myocytes were taken on a Nikon A1R confocal system with NIS-Elements C imaging software.

### Coimmunoprecipitations, Protein Extraction, and Western Blotting

Heart extracts were resolved by SDS-PAGE, transferred to PVDF membranes, and immunoblotted using standard methods [29]. Primary antibodies specific to ERK1/2 (sc-135900), p-ERK1/2 (sc-16981-R), p-p38 (sc-7975-R), p38 (sc-535), p-S727-Stat3 (sc-21876-R), p-Y705-STAT3 (sc-7993), STAT3 (sc-482), PKCα (sc-208), PKCε (sc-214), vinculin (sc-55465), α-tubulin (sc-8035) (all from Santa Cruz), p-S473-Akt (Cell Signaling Technologies, 736E11), Akt, (Cell Signaling Technologies, 11E7), myosin heavy chain (MF20, Developmental Studies), GAPDH (Millipore, MAB374), α-sarcomeric actin (Sigma, clone 5C5), α-actinin (Sigma, clone EA-53), γ-actin (Millipore, AB3625), β-actin (Abcam, ab6276), talin (Sigma, clone 8d4), integrin β1 (BD Transduction Laboratories, clone 18/cd29), ANF (Peninsula Laboratories, T-4015), myosin binding protein C [26], cardiac troponin T (Thermo Scientific, clone 13–11), cardiac troponin I (Immunochemical, clone 6F9), and LRRC10 [4] were used followed by HRP conjugated secondary antibodies (Santa Cruz). Protein bands were detected by chemiluminesence (Thermo Scientific) and quantified with NIH Image J.

A particulate fraction enriched in myofibril proteins and nonparticulate fraction were extracted from 2–3 month old mouse ventricles for immuoblotting as described previously [16]. Coimmunoprecipitation experiments were performed as described [29]. Briefly, one-two month old hearts were homogenized in IP buffer (20 mM Tris HCl, pH 8.0, 100 mM NaCl, 1 mM EDTA) with 1% triton X-100, 1 mM DTT, and protease inhibitors (1 mM AEBSF (AppliChem), 10 μg/mL leupeptin (Roche), 10 μg/mL aprotinin (Roche)). Cardiac lysates were incubated with preimmune serum or a LRRC10 antibody followed by addition of Protein A agarose beads (Santa Cruz). Immunoprecipitated proteins were immunoblotted.

### Glutathione S-Transferase (GST)-pulldown Assays

To produce the GST-LRRC10 fusion protein, mouse *Lrrc10* was amplified by PCR and inserted in frame into the pGEX-2T vector containing an N-terminal GST (GE Healthcare). GST or GST-LRRC10 were expressed in *E.Coli* BL21 (DE3, Merck) and purified with glutathione agarose (Sigma) as described [31]. Pulldown assays were performed as described [32] with slight modifications. 5 μg of purified rabbit skeletal muscle α-actin (Cytoskeleton, AKL95) or 20 μg of cytoskeletal nonmuscle actin (Cytoskeleton, APHL95, 85% β-actin, 15% γ-actin) was incubated with 1.5 μg GST or GST-LRRC10 for two hours in incubation buffer (PBS, 0.1 mM CaCl_2_, and 1% Triton X-100) followed by washing in wash buffer (20 mM Tris HCl, pH 7.4 and 150 mM NaCl). Bound proteins were immunoblotted.

### Myocardial Contractility and Analysis of Myofibrillar Protein Content

Skinned trabeculae were prepared [33] and mechanical properties were examined [34] as described previously. Analysis of myofibrillar protein content by silver staining was performed as previously described [34]. Active force-pCa and *k*_tr_-pCa/relative force relationships were established at sarcomere length (SL) of ∼2.20 μm [34]. Steady-state force and the apparent rate constant of force redevelopment (*k*_tr_) were measured simultaneously using the modified multi-step protocol developed by Brenner and Eisenberg [34], [35]. To determine the Ca^2+^ sensitivity of isometric force (pCa_50_), the force-pCa data were fitted with the Hill equation: P/P_o_  =  [Ca^2+^]^n^/(*k* ^n^ + [Ca^2+^]^n^)], where n is slope (Hill coefficient) and *k* is the Ca^2+^ concentration required for half-maximal activation (pCa_50_). Apparent rate constants of force redevelopment (*k*_tr_) were determined by linear transformation of the half-time of force recovery (*k*_tr_  = –ln 0.5(t_1/2_)*^−^* ^1^), as described previously [34], [36].

### Quantitative Real Time PCR, Microarray Analyses and Statistical Analysis

Quantitative real-time PCR (qRT-PCR) was performed using FastStart SYBR Green Master (Roche) on a BioRad iCycler as described [37]. All samples were assayed in duplicate with nearly identical replicate values. Data were generated using the standard curve method and normalized to 18S expression. All primers were thoroughly evaluated by melt curve analysis to ensure the amplification of a single, desired amplicon.

Microarray analysis was performed using a *Mus musculus* Whole Mouse Genome Array (4×44K, Agilent Technologies) as described [37], [38]. RNA isolated (QIAzol, Qiagen) from two wildtype (WT) or *Lrrc10^−^* ^/*−*^ littermate hearts at E15.5 or two months of age was pooled and microarray analysis was performed on three independent biological replicates. Data from the scan file was analyzed using EDGE^3^ software [38] and generated data is uploaded on the NCBI GEO (Gene Expression Omnibus) database (GSE40600 and GSE36508). Dysregulated genes were further sorted into KEGG biological pathways using DAVID functional analysis [39].

All results are expressed as mean ± standard error of the mean. All statistical analysis was performed by a student's t-test unless otherwise stated. P-value <0.05 was considered statistically significant. ^*^p<0.05, ^**^p<0.01, ^***^p<0.001.

## Results

### Genetic ablation of Lrrc10 Results in Dilated Cardiomyopathy

**Figure 1:**
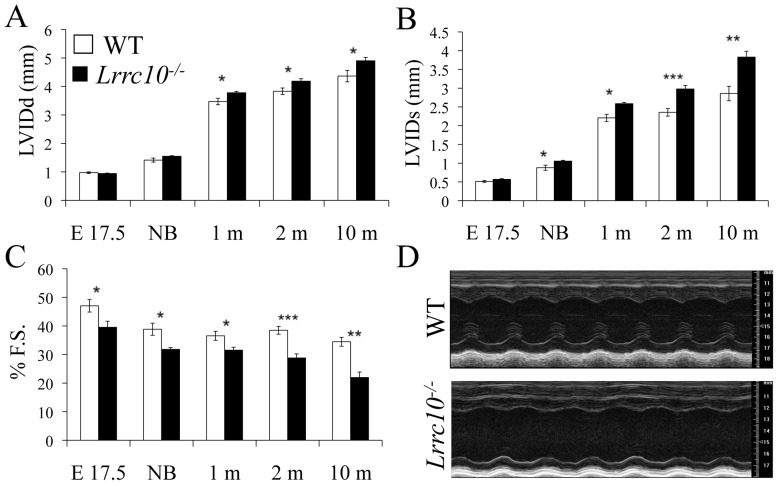
*Lrrc10^−/−^* mice develop dilated cardiomyopathy. Echocardiography reveals cardiac functional deficits in *Lrrc10* ***^−^*** ^*/****−***^ mice. Left ventricular inner diameter (LVID) measurements during (A) diastole and (B) systole and (C) fractional shortening (F.S.) in WT and *Lrrc10* ***^−^*** ^*/****−***^ mice at the indicated age. (D) Representative M-mode images at 10 months of age. *E*, embryonic day; *NB*, newborn; *m*, months; *d*, diastole; *s*, systole. n = 4–13.

LRRC10 shows remarkable cardiac-specific expression from precardiac mesoderm to cardiomyocytes in adult hearts [4], [7]. To determine the function of LRRC10 in the adult mammalian heart, cardiac function of *Lrrc10^−/−^* mice was analyzed at various ages by echocardiography (Fig 1, Table 1). Postnatal *Lrrc10^−/−^* mice exhibit left ventricular (LV) chamber dilation and systolic dysfunction as evidenced by significant increases in LV inner diameter at end diastole (LVIDd) and end systole (LVIDs) (Fig 1A, 1B), respectively, within one month of age. LV dilation in adult *Lrrc10^−/−^* mice was not accompanied by a change in interventricular septal (LVAW) or LV posterior wall (LVPW) thickness (Table 1). A decrease in fractional shortening (%FS) was detected in *Lrrc10^−/−^* hearts at all stages examined (Fig 1C), implying defective cardiac function during perinatal and postnatal life. M-mode images depict systolic dysfunction and ventricular dilation in aged adult *Lrrc10^−/−^* hearts (Fig 1D), hallmark features of DCM [2]. No alterations in heart rate (Table 1) or mitral and aortic valve function (data not shown) were detected in *Lrrc10^−/−^* hearts, suggesting that the observed cardiac functional deficits are due to systolic dysfunction. Interestingly, impairment of systolic function in *Lrrc10^−/−^* hearts was observed in utero as early as embryonic day (E) 17.5 (Fig 1C), prior to the development of DCM. Postnatal *Lrrc10^−/−^* hearts proceed to a dilated phenotype without undergoing concentric hypertrophic growth or LV wall thickening (Table 1). In summary, *Lrrc10^−/−^* mice exhibit early-onset cardiomyopathy that develops into DCM by one month of age, with a progressive decline in cardiac function.

**Table 1:** Echocardiographic assessment of cardiac structure and function in *Lrrc10^−/−^* mice.

	E 17.5		newborn		1 month		2 months		10 months	
	WT	*Lrrc10^−/−^*	WT	*Lrrc10^−/−^*	WT	*Lrrc10^−/−^*	WT	*Lrrc10^−/−^*	WT	*Lrrc10^−/−^*
**N**	13	12	11	11	6	6	6	7	4	5
**LVID; d (mm)**	0.97±.03	0.94±.02	1.42±.07	1.55±.03	3.47±.12	3.78±.05*	3.83±.12	4.18±.09*	4.36±.20	4.90±.12*
**LVID; s (mm)**	0.51±.03	0.57±.02	0.88±.07	1.06±.02*	2.21±.10	2.59±.03**	2.36±.10	2.98±.10***	2.86±.19	3.83±.16**
**LVPW; d (mm)**			0.28±.01	0.28±.01	0.58±.01	0.50±.03	0.70±.02	0.69±.02	0.76±.01	0.78±.06
**LVPW; s (mm)**			0.47±.01	0.48±.01	0.99±.02	0.82±.04*	1.23±.02	1.08±.05*	1.16±.04	1.05±.07
**LVAW; d (mm)**	0.30±.01	0.31±.01	0.29±.01	0.28±.01	0.57±.01	0.53±.03	0.72±.03	0.69±.02	0.75±.03	0.72±.04
**LVAW; s (mm)**	0.45±.02	0.41±.02	0.49±.02	0.47±.01	0.95±.04	0.89±.04	1.31±.03	1.04±.05***	1.28±.09	1.08±.06
**LV Volume; d (μL)**	0.95±.09	0.85±.04	5.49±.67	6.64±.20	50.35±3.97	61.34±1.74*	63.60±4.54	78.29±3.70*	86.79±9.36	113.33±6.24*
**LV Volume; s (μL)**	0.15±.03	0.19±.02	1.66±.30	2.46±.09*	16.60±1.72	24.37±.77**	19.75±1.97	34.85±2.84***	32.06±5.12	63.76±6.13**
**% E.F.**	84.24±1.90	77.08±2.74*	72.36±2.51	63.05±.76*	67.19±2.04	60.01±1.42*	69.18±1.73	55.58±2.16***	63.65±2.23	44.04±3.31**
**% F.S.**	47.03±.02	39.54±.02*	38.84±2.12	31.81±.64*	36.54±1.57	31.54±.98*	38.48±1.38	28.78±1.41***	34.45±1.56	21.96±1.89**
**LV Mass (mg)**	3.24±.21	3.37±.13	5.51±.43	6.01±.13	60.51±4.58	60.07±3.89	93.96±5.25	104.97±5.01	125.63±11.03	160.18±20.01
**LV Mass/BW (mg/g)**			4.22±.35	4.56±.11	4.54±.28	4.88±.25	4.07±.23	4.65±.25	3.32±.14	4.96±.44*
**Heart Rate (bpm)**	243±10	248±6	419±13	418±9	492±17	536±13	450±36	494±17	478±18	484±29
**Body Weight (g)**			1.31±0.01	1.32±0.03	13.69±1.51	12.32±0.59	23.15±0.62	22.66±0.69	30.51±4.93	31.95±1.28

Evaluation of cardiac structural and functional parameters by echocardiography in *Lrrc10* ^−*/*−^ and control (WT) mice at various ages. *LV*, left ventricle; *ID*, inner diameter; *PW*, posterior wall; *AW*, anterior wall; *d*, diastole; *s*, systole; *E.F.* ejection fraction; *F.S.* fractional shortening.

**Figure 2:**
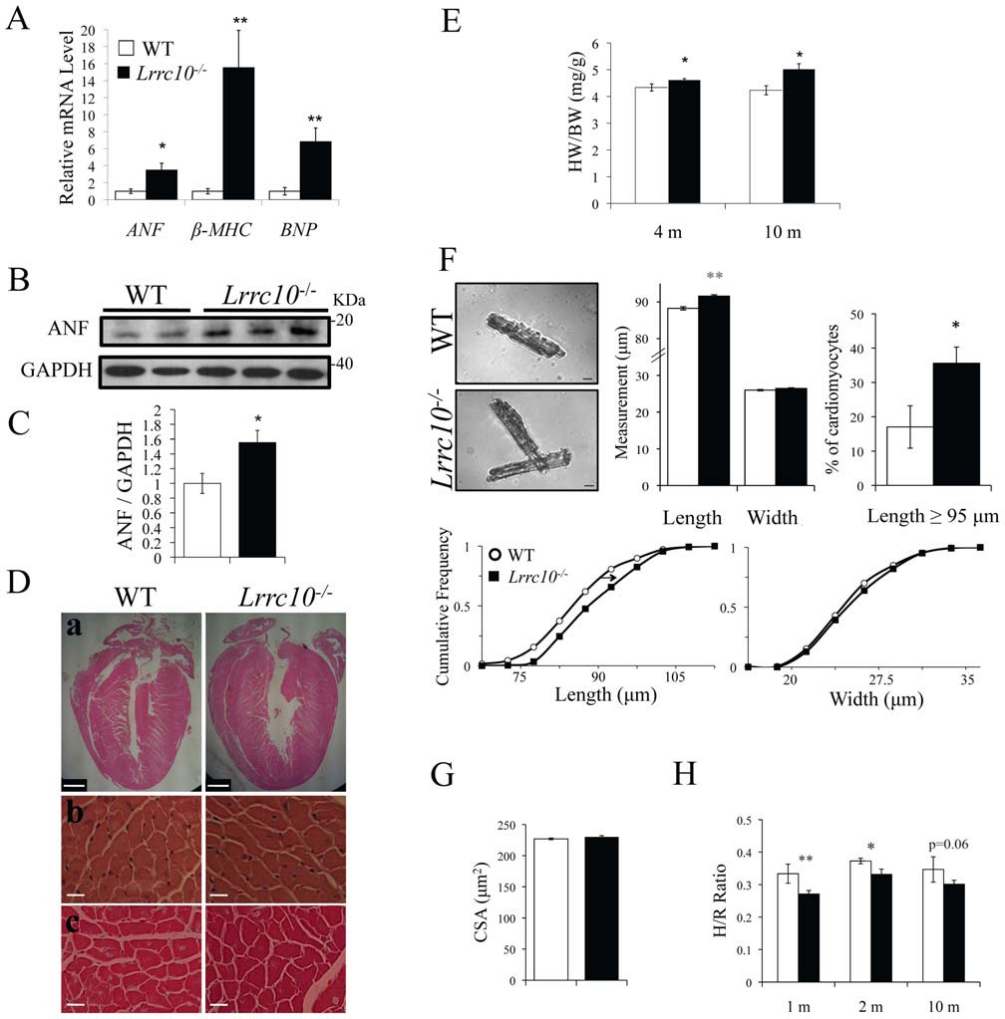
Characterization of *Lrrc10^−/−^* hearts. (A) qRT-PCR analysis for *ANF* (*Nppa*), *β-MHC* (*Myh7*), and *BNP* (*Nppb*) demonstrates re-expression of the fetal genes in *Lrrc10* ***^−^*** ^*/****−***^ hearts at 4 months of age (n = 7–8). (B) Representative immunoblot and (C) quantitation of ANF expression in WT and *Lrrc10* ***^−^*** ^*/****−***^ hearts (n = 4). Expression level was normalized to GAPDH as a loading control. (D) Histology of WT and *Lrrc10* ***^−^*** ^*/****−***^ mice at ten months of age. Representative (a) frontal midline heart sections (scale bar = 1 mm) and (b) 40X high magnification images (scale bar = 20 μm) of H&E and (c) Masson's trichrome staining. (E) Wet heart weight to body weight ratios indicate increased size of *Lrrc10* ***^−^*** ^*/****−***^ hearts in adulthood (n = 6–12). (F) Isolated adult WT and *Lrrc10* ***^−^*** ^*/****−***^ ventricular cardiomyocytes were micrographed (*top left,* scale bar = 10 μm) to measure cell length and width (n = 384 cells from 3 WT hearts and 435 cells from 3 *Lrrc10* ***^−^*** ^*/****−***^ hearts). (G) Cardiomyocyte cross-sectional area (CSA) in WT and *Lrrc10* ***^−^*** ^*/****−***^ hearts at 10 months (n = 4–6). (H) To measure relative left ventricle wall thickness as compared to left ventricle size, H/R ratio was calculated from echocardiography data. H/R ratio  =  (LVPWd+LVAWd)/LVIDd. *m*, months.

To evaluate pathological cardiac remodeling, transcript levels of molecular markers of cardiac hypertrophy/failure were determined by qRT-PCR. *Lrrc10^−/−^* hearts exhibit significant induction of the fetal genes *ANF* (atrial natriuretic factor, *Nppa*), *β-MHC* (β-myosin heavy chain, *Myh7*), and *BNP* (brain natriuretic peptide, *Nppb*) (Fig 2A), indicating pathological cardiac hypertrophy. Upregulation of ANF in *Lrrc10^−/−^* hearts was confirmed at the protein level by Western blotting (Fig 2B, 2C).

*Lrrc10^−/−^* mice exhibit overtly normal cardiac morphology throughout development [25] (Fig 2D) and have a normal lifespan without increased mortality (data not shown). However, adult *Lrrc10^−/−^* mice exhibit enlarged hearts compared to wild type (WT) at four and ten months of age (Fig 2Da, 2E). Modest cardiac enlargement without significant changes in ventricular wall thickness was observed by histology in aged *Lrrc10^−/−^* mice (Fig 2Da) consistent with echocardiography data (Table 1). Wet heart weight-to-body weight ratios are increased in *Lrrc10^−/−^* mice by four months of age (Fig 2E), suggesting pathological cardiac growth. To evaluate cardiomyocyte remodeling in *Lrrc10^−/−^* hearts, cell length and width were measured in isolated ventricular myocytes at three months of age. Morphometric data indicate an increase in length of *Lrrc10^−/−^* cardiomyocytes without a change in cell width (Fig 2F), suggesting eccentric cardiac growth in *Lrrc10^−/−^* mice. No changes in cardiomyocyte cross-sectional area were detected in *Lrrc10^−/−^* hearts at four or ten months of age (Fig 2G, data not shown), confirming a lack of concentric cardiac hypertrophy. To quantitatively assess concentric hypertrophy versus eccentric cardiac growth or dilation, relative LV wall thickness (the ratio of LV wall and septal thickness to LV chamber radius or “H/R ratio”) was analyzed in WT and *Lrrc10^−/−^* mice. Reduced H/R ratios in adult *Lrrc10^−/−^* mice confirm dilation or eccentric cardiac growth (Fig 2E).

Histological analyses indicate a lack of gross myocyte disarray (Fig 2Db) or significant fibrosis by Masson's trichrome staining in *Lrrc10^−/−^* hearts at one, two (data not shown), and ten months of age (Fig 2Dc). No significant increases in mitosis were detected in embryonic or adult *Lrrc10^−/−^* heart sections by phosphohistone-H3 immunostaining (Figure S1), consistent with previous reports [25]. Alterations in apoptosis were not detected in *Lrrc10^−/−^* hearts at two and ten months of age by TUNEL (data not shown). Results indicate that cardiac enlargement in *Lrrc10^−/−^* mice is not due to differences in cardiomyocyte proliferation or apoptosis.

### LRRC10 interacts with α-actinin and actin

**Figure 3:**
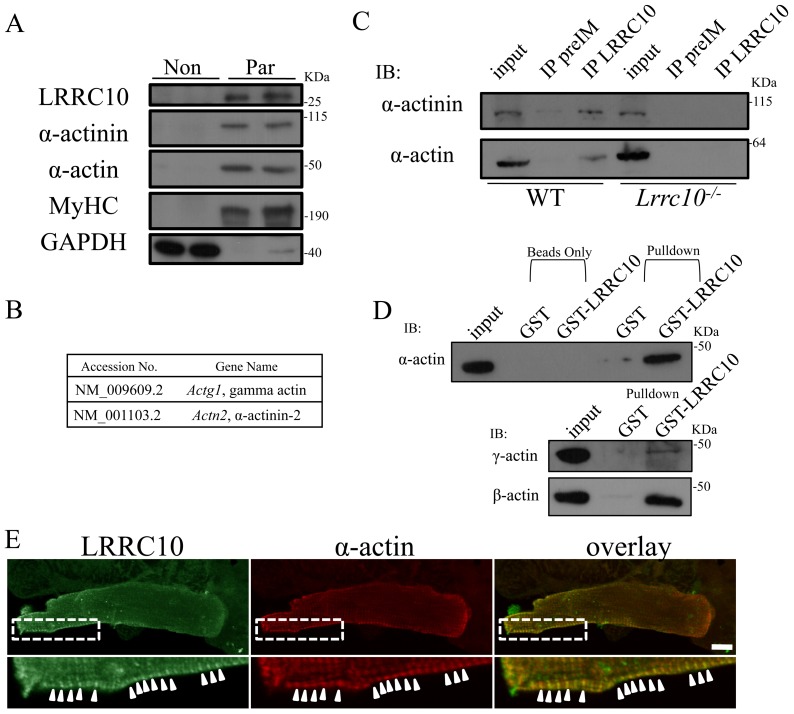
LRRC10 interacts with actin and α-actinin. (A) LRRC10 cofractionates with α-actin and α-actinin in the particulate fraction. Heart extracts from 10-week-old mice were fractionated into a nonparticulate fraction (Non) and a particulate fraction (Par) enriched in myofibril proteins. GAPDH and myosin heavy chain (MyHC) were used as nonparticulate and particulate controls, respectively. (B) Yeast-two hybrid screening identified α-actinin and γ-actin as binding partners of LRRC10. (C) α-actinin and α-actin endogenously interact with LRRC10 in the heart. WT or *Lrrc10* ***^−^*** ^*/****−***^ mouse heart extracts were immunoprecipitated (IP) with preimmune serum (PreIM) or an LRRC10 antibody and immunblotted for α-actinin or α-actin. (D) LRRC10 interacts with α-, β-, and γ-actin in vitro. Purified α-actin or cytoskeletal actin was incubated with GST or a GST-LRRC10 fusion protein and pulled down proteins were immunoblotted for α-actin, γ-actin, or β-actin. (E) LRRC10 colocalizes with α-actin. Coimmunostaining for LRRC10 and α-actin in adult mouse ventricular cardiomyocytes. Enlargement of boxed area is shown at *bottom*. Scale bar  = 10 μm.

To gain greater insight into the subcellular location of LRRC10, fractionation experiments were performed using ten week old hearts in which a particulate fraction enriched with myofilament proteins was separated from a nonparticulate soluble fraction. Interestingly, LRRC10 fractionates almost entirely with the particulate fraction (Fig 3A), suggesting that LRRC10 is tightly associated with myofilaments and/or nonsoluble membranous components and is not freely soluble in the cardiomyocyte cytoplasm. α-actin, the sarcomeric actin isoform that constitutes the cardiac actin thin filaments, localizes entirely to the particulate fraction as expected, and so do α-actinin and myosin heavy chain (MyHC). In contrast, GAPDH was found in the nonparticulate fraction (Fig 3A).

LRRC10 contains no known functional domains other than its seven LRRs [4], [5], [7], which are known protein-protein interaction motifs [8]. We therefore hypothesized that LRRC10 mediates functions critical for cardiac biology via physical interactions with other proteins. To identify potential proteins that physically interact with LRRC10, we performed yeast two hybrid screening. Among the fourteen positive clones identified were α-actinin and γ-actin (Fig 3B). α-actinin crosslinks actin thin filaments of adjacent sarcomeres at the Z-disc as well as interacts with a number of other proteins crucial to cardiomyocyte function [3], [11]. γ-actin is the costameric actin isoform that exhibits a striated immunostaining pattern and localizes to the cardiomyocyte cytoskeleton at the periphery of the Z-disc [12]. To test if LRRC10 endogenously interacts with α-actinin and actin in the heart, extracts from WT and *Lrrc10^−/−^* hearts were immunoprecipitated with an LRRC10 antibody followed by immunoblotting for α-actinin or α-actin. Our data indicate that α-actinin coimmunoprecipitates with LRRC10 in the adult WT heart (Fig 3C, *top panel*). Because actin isoforms exhibit greater than 90% amino acid sequence homology [40] and γ-actin is a minor component of total cardiac actin, we tested whether LRRC10 endogenously interacts with α-actin in the heart by coimmunoprecipitation. LRRC10 indeed interacts with α-actin in WT heart extracts (Fig 3C, *bottom panel*). α-actin and α-actinin are not immunoprecipitated in *Lrrc10^−/−^* heart extracts (Fig 3C, *right*), demonstrating the specificity of the immunoprecipitation. Therefore, LRRC10 endogenously interacts with α-actinin and α-actin or interacts with a complex containing α-actinin and α-actin in the heart.

To determine if LRRC10 directly binds actin isoforms, GST pulldown assays were performed in vitro. α-actin interacts with GST-LRRC10 but not GST (Fig 3D, *top*), suggesting that LRRC10 directly interacts with α-actin. To evaluate whether LRRC10 interacts with γ-actin as predicted by yeast two-hybrid screening (Fig 3B), pulldown assays were also performed with purified cytoskeletal actin. Results show that LRRC10 also directly interacts with γ-actin (Fig 3D, *middle*). Given that LRRC10 interacts with both α-actin and γ-actin in vitro and the high homology of actin isoform amino acid sequences, we investigated whether LRRC10 interacts with β-actin, the other cytoskeletal actin isoform. Pulldown assays show that LRRC10 and β-actin interact (Fig 3D, *bottom*), suggesting the possibility that LRRC10 is a pan-actin binding protein.

Since intracellular location of proteins can provide critical information on physical interactions as well as potential function, we examined the expression pattern of endogenous LRRC10 in adult mouse ventricular myocytes. Our previous studies have shown that LRRC10 colocalizes with α-actinin in adult cardiomyocytes [4]. Immunostaining of α-actin also exhibits striations that colocalize with LRRC10 in adult cardiomyocytes (Fig 3E), providing supporting evidence that LRRC10 and α-actin interact in the heart. No differences in expression pattern were detected for α-actin and α-actinin in adult *Lrrc10^−/−^* cardiomyocytes by immunostaining (data not shown), suggesting that LRRC10 is not required to maintain sarcomeric integrity or for proper localization of α-actin or α-actinin.

### Lrrc10^−/−^ Hearts Exhibit Defective Gene Expression

To identify early perturbations in gene expression in the *Lrrc10^−/−^* heart at the onset of cardiac dysfunction but prior to the development of DCM in adulthood, microarray analysis was conducted on embryonic hearts at E15.5. Genes consistently up- or downregulated in *Lrrc10^−/−^* hearts across all three biological replicates were analyzed using DAVID functional analysis software [39] to identify defective pathways.

**Figure 4:**
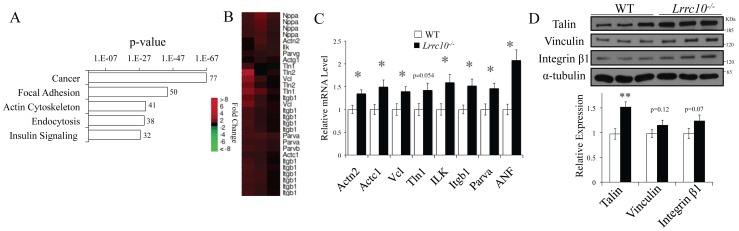
Gene expression profiling of embryonic *Lrrc10^−/−^* hearts. Microarray analysis was performed on WT and *Lrrc10* ***^−^*** ^*/****−***^ hearts at E15.5 to identify dysregulated pathways in the absence of *Lrrc10*. (A) Pathway analysis on genes upregulated 1.2-fold or greater (p<0.05, signal ≥20) at E15.5. Shown are the five most significantly upregulated KEGG pathways in *Lrrc10* ***^−^*** ^*/****−***^ hearts using DAVID. The number at the end of the histograms represents the number of upregulated genes in the KEGG pathway. (B) Heat maps from expression profiling and (C) qRT-PCR analyses demonstrate upregulation of actin cytoskeloton transcripts in embryonic *Lrrc10* ***^−^*** ^*/****−***^ hearts (n = 5–10). (D) Western blotting analysis of cytoskeletal protein expression in E15.5 hearts (n = 4–5). *Actn2*, actinin alpha 2; *Actc1,* actin alpha, cardiac muscle 1;*Vcl*, vinculin; *Tln1*, talin 1; *ILK*, integrin-linked kinase; *Itgb1*, integrin β1; *Parva*, parvin alpha; *E*; embryonic day.

Pathway analysis of gene expression data in embryonic *Lrrc10^−/−^* hearts identified regulation of the actin cytoskeleton and focal adhesion as among the most upregulated KEGG pathways (Fig 4A). Expression profiling indicates upregulation of α-actinin (*Actn2*), α-actin (*Actc1*), and γ-actin (*Actg1*) as well as a number of other cytoskeletal genes (Fig 4B). qRT-PCR analyses confirm upregulation of α-actinin and α-actin transcripts in embryonic *Lrrc10^−/−^* hearts (Fig 4C) as well as critical cardiac cytoskeletal genes including vinculin (*Vcl*), integrin-linked kinase (*ILK*), integrin β1 (*Itgb1*), and parvin α (*Parva*) (Fig 4C). Moreover, ANF (*Nppa*) expression, which is upregulated in cardiomyocytes in response to biomechanical stress [41], is significantly elevated in the embryonic *Lrrc10^−/−^* heart (Fig 4B, 4C). Western blotting of cardiac extracts at E15.5 demonstrates increased talin and a trend towards increased vinculin and integrin β1 expression at the protein level in *Lrrc10^−/−^* hearts (Fig 4D), indicating increased abundance of critical cardiac cyotskeletal proteins in the absence of LRRC10.

**Figure 5:**
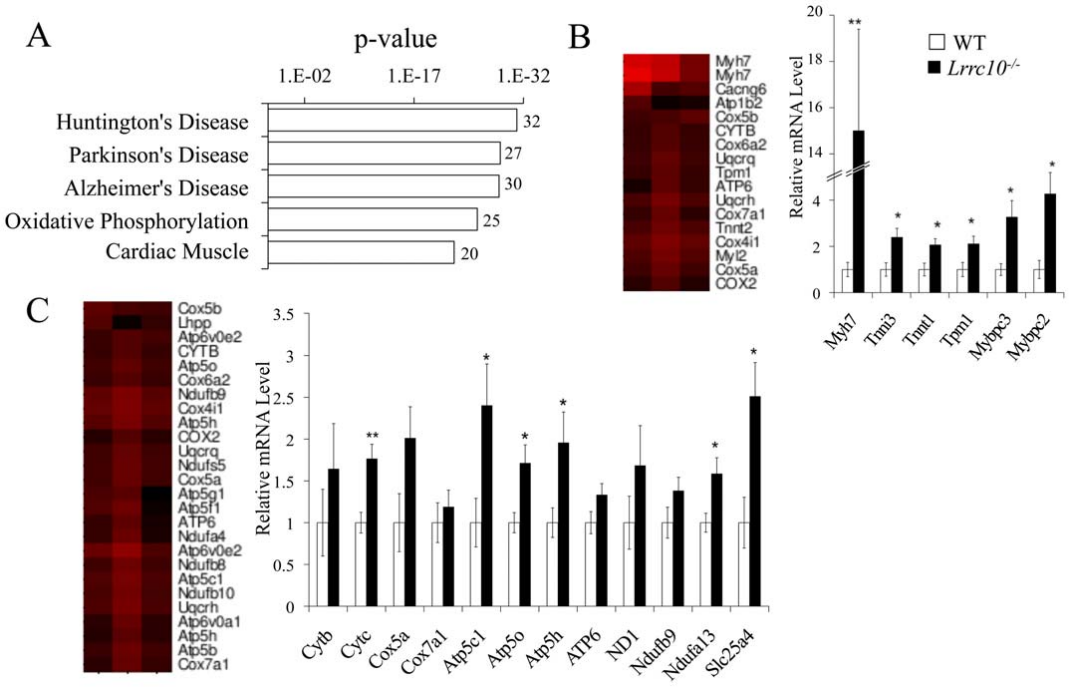
Gene expression profiling of adult *Lrrc10^−/−^* hearts. Microarray analysis was performed on WT and *Lrrc10* ***^−^*** ^*/****−***^ hearts at two months of age to identify dysregulated pathways in the absence of *Lrrc10*. (A) Pathway analysis on genes upregulated 1.2-fold or greater (p<0.05, signal ≥20) at two months of age. Shown are the five most significantly upregulated KEGG pathways in *Lrrc10* ***^−^*** ^*/****−***^ hearts using DAVID. The number at the end of the histograms represents the number of upregulated genes in the KEGG pathway. (B, C) Heat maps from expression profiling (*left*) and qRT-PCR analyses (*right,* n = 6–12) demonstrate upregulation of many (B) cardiac muscle contraction and (C) oxidative phosphorylation transcripts in adult *Lrrc10* ***^−^*** ^*/****−***^ hearts. *Myh7*, β-myosin heavy chain; *Tnni3*, troponin I, cardiac 3; *Tnnt1*, troponin T1, skeletal, slow; *Tpm1*, tropomyosin 1, alpha; *Mybpc3,* myosin binding protein C, cardiac; *Mybpc2*, myosin binding protein C, fast-type; *Cytb*, cytochrome b; *Cytc*, cytochrome c; *Cox5a*, cytochrome c oxidase subunit Va; *Cox7a1*, cytochrome c oxidase subunit VIIa1; *Atp5c1*, ATP synthase, H+ transporting, mitochondrial F1 complex, gamma polypeptide 1; *Atp5o*, ATP synthase, H+ transporting, mitochondrial F1 complex, O subunit; *Atp5h*, ATP synthase, H+ transporting, mitochondrial F0 complex, subunit d; *ATP6,* ATP synthase F0 subunit 6; *ND1*, NADH dehydrogenase subunit 1; *Ndufb9*, NADH dehydrogenase (ubiquinone) 1 beta subcomplex, 9; *Ndufa13*, NADH dehydrogenase (ubiquinone) 1 alpha subcomplex, 13; *Slc25a4*, solute carrier family 25 (mitochondrial carrier, adenine nucleotide translocator), member 4.

To determine molecular alterations that occur during the pathogenesis of DCM in adult *Lrrc10^−/−^* hearts, microarray analyses were also performed at two months of age. Adult *Lrrc10^−^/^−^* hearts exhibited enrichment of upregulated transcripts from the dilated cardiomyopathy KEGG pathway (data not shown). Clustering of microarray gene expression data confirmed β-MHC (*Myh7*) and *ANF* as among the most significantly upregulated genes in adult *Lrrc10^−^/^−^* hearts (Fig 2A, data not shown). However, the pathways undergoing the most statistically significant dysregulation in the adult *Lrrc10^−^/^−^* heart were oxidative phosphorylation and cardiac muscle contraction (Fig 5A), prompting further investigation of transcript levels in these pathways. Upregulation of cardiac muscle contraction genes was confirmed in *Lrrc10^−^/^−^* hearts, including isoforms of troponin T, troponin I, and myosin binding protein C (Fig 5B). qRT-PCR analyses confirmed the upregulation of genes involved in oxidative phosphorylation, including multiple subunits of cytochrome c oxidase, NADH dehydrogenase, and ATP synthase (Fig 5C). Pathway analysis of embryonic and adult *Lrrc10^−/−^* hearts did not identify any biologically relevant downregulated pathways at high level of statistical significance (data not shown).

Modest global changes in gene expression were observed at both stages with more than half of genes dysregulated 1.2-fold or greater across all three chips having average changes in gene expression between 1.2- and 1.4-fold. Thus, enrichment of upregulated transcripts in the oxidative phosphoryation and cardiac muscle contraction pathways in adult and actin cytoskeleton pathway in embryonic *Lrrc10^−/−^* hearts represents significant changes in gene expression. Taken together our data show that prenatal *Lrrc10^−/−^* hearts exhibit different global changes in gene expression compared to adult hearts (Fig 4 vs. Fig 5), suggesting that early transcriptional alterations of actin cytoskeletal components may be directly linked to the molecular function of LRRC10 while gene expression changes in adulthood are likely to be compensatory or associated with the progression of DCM.

### Lrrc10^−/−^ Hearts Exhibit Defective Signal Transduction

**Figure 6:**
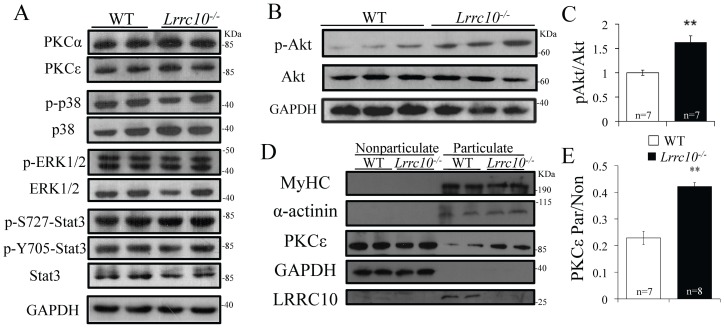
Signal transduction in *Lrrc10^−/−^* hearts. (A) Western blotting analysis for activation of Akt, p38, ERK, and STAT signaling pathways and PKC expression reveals no changes in phosphorylation of p38, ERK, or STAT or protein abundance of PKCα or PKCε. (B) Western blotting indicates hyperphosphorylation of Akt at Ser 473 in *Lrrc10* ***^−^*** ^*/****−***^ hearts. (C) Quantitation of Western blotting confirms increased phosphorylation of Akt in *Lrrc10* ***^−^*** ^*/****−***^ hearts (n = 7). Phosphorylated Akt was normalized to total Akt expression and GAPDH was used as a loading control. (D) Representative Western blotting of particulate and nonparticulate fractions of adult heart extracts in control (WT) and *Lrrc10* ***^−^*** ^*/****−***^ mice. (E) Quantification of PKCε expression in the particulate fraction relative to the nonparticulate fraction. Myosin heavy chain (MyHC) and α-actinin were used as a particulate/myofilament loading control.

Alterations in intracellular signal transduction frequently occur in DCM [3]. In an effort to determine molecular defects in *Lrrc10^−/−^* hearts, various signaling pathways known to mediate cardiac hypertrophy were investigated using WT and *Lrrc10^−/−^* heart extracts at 2–3 months of age. Western blotting analyses were performed using antibodies against the p38 and ERK MAPKs, Akt, STAT3, and PKC isoforms. Results indicate no change in the total amount of PKCα or PKCε and no alterations in abundance or phosphorylation status of p38, ERK, or STAT3 in *Lrrc10^−/−^* hearts (Fig 6A). However, *Lrrc10^−/−^* hearts display increased phosphorylation of Akt at Ser 473 without alterations in total Akt abundance (Fig 6B, 6C), suggesting elevated Akt signaling in *Lrrc10^−/−^* hearts.

PKCε is a critical mechanosensing molecule in cardiomyocytes [42]. Activated PKCε has been shown to translocate to the Z-disc and T-tubule in response to a variety of stimuli, resulting in increased abundance in the particulate fraction relative to the cytosolic fraction of heart extracts [43], [44], [45], [46]. Therefore, to gain insight into translocation and activation of PKCε, cardiac extracts were separated into particulate and nonparticulate fractions (Fig 6D). Immunoblotting for PKCε illustrates markedly enhanced translocation to the particulate fraction in *Lrrc10^−/−^* hearts (Fig 6D, 6E), suggesting PKCε activation. Normal localization of actinin and MyHC to the myofilament-enriched particulate fraction and GAPDH in the cytosolic fraction was detected in both control and *Lrrc10^−/−^* hearts (Fig 6D). Therefore, adult *Lrrc10^−/−^* hearts exhibit elevated Akt and PKCε signaling.

### Lrrc10^−/−^ trabecular myocardium does not exhibit significant defects in force development

**Figure 7:**
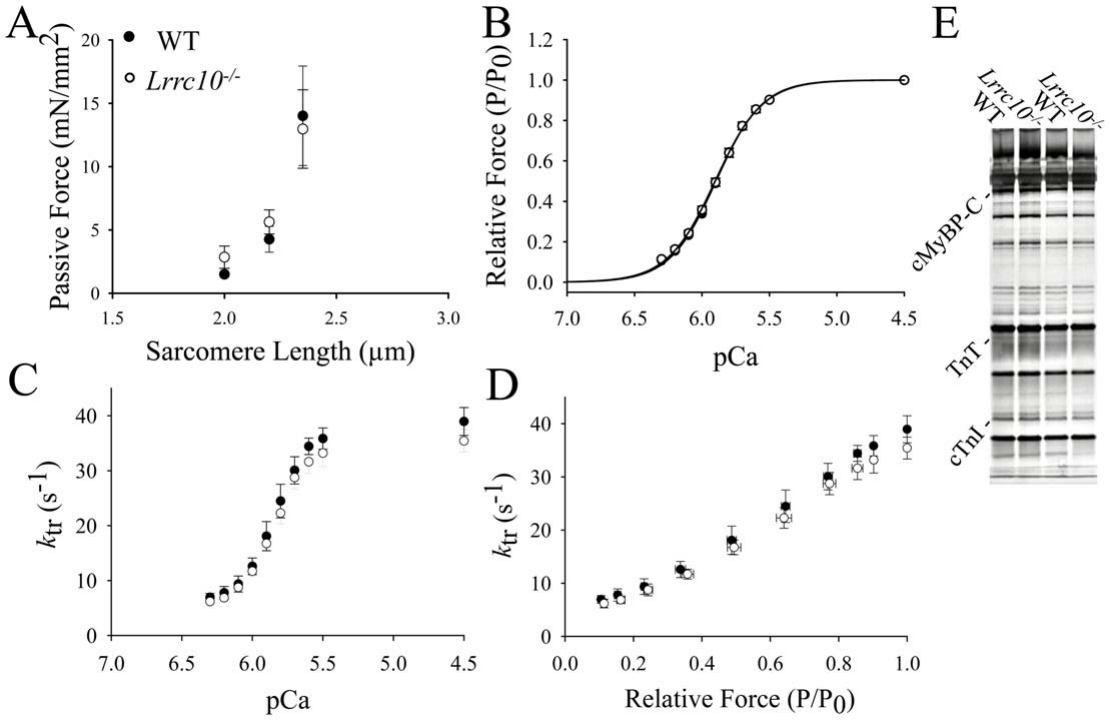
Contractility of skinned myocardium and myofibril protein composition are not altered in *Lrrc10^−/−^* mice. (A) SL-dependent increase in passive force is unaltered in *Lrrc10* ***^−^*** ^*/****−***^ skinned trabeculae. The passive forces were measured in pCa 9.0 first at SL 2.00 μm then at 2.22 and finally at 2.35 μm in WT (n = 4; closed circles) and *Lrrc10* ***^−^*** ^*/****−***^ (n = 4; open circles) skinned trabeculae. (B) Force-pCa relationships established at SL 2.20 μm yielded nearly identical sigmoidal plots in WT and *Lrrc10* ***^−^*** ^*/****−***^ skinned trabeculae. (C–D) Deletion of *Lrrc10* has no effect on the rate of force redevelopment. (C) *k*_tr_-pCa and (D) *k*_tr_-relative force relationships were established at SL 2.22 μm in skinned trabeculae. (E) Silver staining for myofibril protein composition in WT and *Lrrc10* ***^−^*** ^*/****−***^ hearts.

To determine whether LRRC10 has a direct effect on myofilament force development, myocardial contractility was evaluated in skinned trabeculae of three months old WT and *Lrrc10^−/−^* mice. Analyses revealed no significant difference in sarcomere length (SL)-dependent increase in passive force generation (Fig 7A), Ca^2+^ sensitivity of force or apparent cooperativity in activation of force (Fig 7B) in the absence of *Lrrc10*. Force-pCa relationships at SL 2.20 μm were nearly identical in WT and *Lrrc10^−/−^* skinned trabeculae (Fig 7B), yielding pCa_50_  = 5.90 for both control and *Lrrc10^−/−^* trabeculae and Hill coefficients (n_H_) of 2.54 and 2.46 for control and *Lrrc10^−/−^* trabeculae, respectively.

Therefore, passive force generation at a given sarcomere length is similar in control and *Lrrc10^−/−^* myocardium and myofilament calcium sensitivity is unaltered by *Lrrc10* deletion. The apparent rate of force redevelopment was not significantly different in *Lrrc10^−/−^* skinned trabeculae (Fig 7C, 7D), suggesting that deletion of *Lrrc10* does not alter cross-bridge cycling kinetics. Furthermore, deletion of *Lrrc10* had no significant effect on the expression of several myofibrillar proteins as detected by silver staining (Fig 7E), suggesting that alterations in myofilament protein composition are not responsible for systolic dysfunction observed in *Lrrc10^−/−^* hearts (Fig 1). No significant differences in expression of α-actinin, α-actin, myosin binding protein C (Mybpc), cardiac troponin T (cTnT), or cardiac troponin I (cTnI) were detected between WT and *Lrrc10^−/−^* hearts by Western blotting (Figure S2). Therefore, although LRRC10 is required to maintain proper cardiac contractile function, it does not directly regulate myofilament cross-bridge cycling kinetics.

## Discussion

DCM is by far the most common form of pediatric cardiomyopathy, representing 50–60% of all childhood cardiomyopathies [47], [48]. However, the molecular pathways leading to DCM remain largely unknown. Here, we demonstrate that *Lrrc10^−/−^* mice exhibit systolic dysfunction in utero that develops into DCM in postnatal life with progressive deterioration of cardiac function in adulthood. Deletion of a number of genes with critical roles in cardiac physiology does not result in cardiac defects at baseline, but shows the development of DCM in response to biomechanical stress [49], [50]. Notably, *Lrrc10^−/−^* mice develop DCM without pathological stress, indicating a crucial role of LRRC10 in proper cardiac function.

We demonstrate that LRRC10 physically interacts with α-actin and α-actinin in the heart and directly interacts with all actin isoforms in vitro. Disturbance of the mechanical link between the Z-disc and the sarcolemma and/or cytoskeletal proteins has been shown to cause DCM [3], [16]. α-actinin binds a plethora of Z-disc and costameric proteins and mutations in α-actinin are associated with DCM in humans [51]. γ-actin forms a mechanical linkage that stabilizes the sarcomere from mechanical stress in muscle [52] and mutations in cardiac α-actin cause DCM in humans [53] and mice [54]. The LRR domain of tropomodulin-1 is responsible for its localization to the pointed ends of actin filaments at the Z-disc [55] and the testis-specific LRRC67 interacts with α-, β-, and γ-actin to form a cyotoskeletal complex [56], suggesting that the LRR motifs can serve as an actin binding domain. Further, deletion of enigma homologue protein (ENH) results in DCM, likely by disruption of a costameric complex that links the Z-disc to the sarcolemma by interacting with α-actinin and filamin C [16]. In addition to structural roles, Z-disc associated proteins and LRRCs play a role in intracellular signal transduction. LRRC39 regulates serum response factor-mediated signaling in cardiomyoctes [10] and LRRC67 interacts with and regulates the activity of protein phosphatase 1 (PP1), thereby providing a platform to properly localize signaling molecules to the actin cytoskeleton [56], [57]. Thus, it is plausible that LRRC10 serves as a biomechanical link between the sarcomere and actin cytoskeleton and may transduce critical signals in response to mechanical stress or other stimuli.

Microarray analyses identified upregulation of a number of cytoskeletal transcripts in prenatal *Lrrc10^−/−^* hearts, prior to ventricular dilation, suggesting early defects in actin cytoskeletal function. Embryonic *Lrrc10^−/−^* hearts also upregulate the mechanosensitive gene, *ANF*, as well as *integrin-β1*, *ILK*, and *parvin-α*. Likewise, *Integrin-β1* (*Itgb1*) transcripts [58] and ILK, parvin-α [59], and integrin-β1D [58], [60] protein levels are increased in the heart in response to biomechanical stress.

In contrast, microarray analyses of adult *Lrrc10^−/−^* hearts indicate that oxidative phosphorylation is the most significantly upregulated pathway. Global gene expression profiling of human DCM has identified oxidative phosphorylation as a significantly upregulated pathway [61], [62], [63], including upregulation of the TCA cycle and ATP synthesis pathways, suggesting that the observed upregulation of oxidative phosphorylation genes may be a general compensatory response in the pathogenesis of some forms of DCM. Despite no significant effect of *Lrrc10* deletion on myofilament contractile kinetics or the composition of myofilament proteins, transcription of multiple myocardial contractile genes was significantly upregulated in adult *Lrrc10^−/−^* hearts. This phenomenon has also been observed in expression profiling of human DCM patients, which also upregulate *TNNI3*, *MYL2, MYBPC, TNNT2*, and *TPM1* [62], [63], [64].

*Lrrc10^−/−^* hearts exhibit eccentric cardiac growth and progress directly to a dilated phenotype without undergoing concentric hypertrophy. Eccentric hypertrophy occurs via addition of sarcomeres in series resulting in longer cardiomyocytes and dilation at the whole organ level. Therefore, eccentric cardiac growth can result in increased organ weight in the absence of increased cell surface area at the individual myocyte level as a consequence of serial addition of sarcomeres and ventricular dilation [65], [66]. In contrast, concentric hypertrophy occurs by addition of sarcomeres in parallel resulting in greater cell surface area at the individual myocyte level and increased LV wall thickness [65], [66].

Analyses of hypertrophic signal transduction pathways in adult *Lrrc10^−/−^* hearts indicate activation of PKCε and Akt. PKCε binds actin [67] and other Z-disc and costameric proteins [68], [69] to mediate mechanosensitive signaling in cardiomyocytes [42], [68]. Overexpression of constitutively active PKCε results in concentric [70] or eccentric hypertrophy and DCM [43], [71] in vivo. However, overexpression of constitutively active PKCε in cardiomyocytes in vitro results in cell elongation [42], [72]. Therefore, PKCε activation may mediate eccentric cardiac growth and remodeling in *Lrrc10^−/−^* hearts. Moreover, *Lrrc10^−/−^* hearts do not exhibit changes in ERK 1/2 activation [73] or STAT3 signaling [74], which is associated with concentric hypertrophy.

Akt is a critical regulator of cardiomyocyte growth [75], [76] that is often activated as a protective response to cardiac stress [75], [76]. Enhanced phosphorylation of Akt at Ser 473 has been observed in other animal models of DCM, including *cTnT* K210 deletion mice [77] and tachypacing-induced DCM in dogs [78], [79], as well as in response to transverse aortic constriction in mice [59], [80]. Increased phosphorylation of Akt at Ser 473 has also been detected in the failing human heart [59], [81].

PKCε interacts with and phosphorylates myofilament proteins in the heart [43]. Upregulation of myofilament transcripts in the *Lrrc10^−/−^* heart may be a response to PKCε activation. Indeed, hearts from PKCε transgenic mice upregulate protein levels of cardiac α-actin, troponin T2, α-tropomyosin, and myosin light chain [82]. Both PKCε [83] and Akt [75], [76], [84] mediate cardioprotective effects in part through phosphorylation of mitochondrial proteins. Upregulation of oxidative phosphorylation transcripts in *Lrrc10^−/−^* hearts could be due to PKCε- or Akt-mediated alterations of mitochondrial function. Importantly, activation of cardioprotective PKCε [46], [82] and Akt [75], [76] signaling may be a mechanism for *Lrrc10^−/−^* hearts to be protected from deleterious apoptosis and fibrosis and avert severe heart failure.

We have previously demonstrated that knockdown of Lrrc10 expression in zebrafish embryos results in cardiac dysfunction and embryonic lethality [6] whereas *Lrrc10^−/−^* mice survive to adulthood and develop DCM. Genetic and functional redundancy in the mammalian heart and/or compensatory mechanisms may account for phenotypic differences observed from germline deletion of *Lrrc10* in mice compared to knockdown in zebrafish. Species differences in phenotypic defects have been reported. Knockdown of Gata5 in zebrafish results in abnormal heart development [85] while *GATA5* homozygous knockout mice lack a discernible cardiac phenotype [86]. Knockdown of *cypher* expression in zebrafish results in pericardial dilation and thin ventricular wall without lethality [87] while deletion of *Cypher* in mice results in DCM and mortality between postnatal days 1 and 5 [88].

Here, we report that LRRC10 is required for proper mammalian cardiac function. *Lrrc10^−/−^* mice exhibit prenatal systolic dysfunction that results in early onset DCM and progressive postnatal cardiac functional deficits. *Lrrc10^−/−^* mice will therefore provide an opportunity to investigate childhood cardiomyopathy.

## Supporting Information

Figure S1 ***Lrrc10^−/−^*** **hearts undergo normal proliferation.** Immunostaining for phosphohistone H3 (p-H3) as a mitotic marker in WT and *Lrrc10^−/−^* heart sections showed no alterations in proliferation at E13.5 or one month of age.
(PDF)

Figure S2 **Myofibril protein expression in** ***Lrrc10^−/−^*** **hearts.** No significant difference in protein expression of myofibril proteins between WT and *Lrrc10^−/−^* hearts at four months of age was detected by Western blotting (n = 3–4) using various antibodies as indicated. *cTnI*, cardiac troponin I; *cTnT*, cardiac troponin T; *Mybpc*, myosin binding protein-C.
(PDF)

Materials and Methods S1 **Supplemental Methods.**
(DOCX)
