# Supplementary figures and images for: Ablation of the Cardiac-Specific Gene Leucine-Rich Repeat Containing 10 (Lrrc10) Results in Dilated Cardiomyopathy
Source: PLoS One. 2012 Dec 7;7(12):e51621. doi: 10.1371/journal.pone.0051621 (PMC3517560; doi:10.1371/journal.pone.0051621)

Supplemental Figure S1.

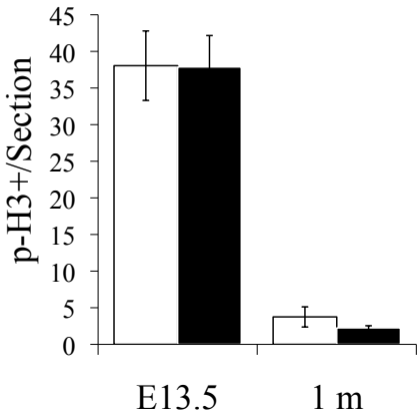

Supplement: Figure S1 — Lrrc10−/− hearts undergo normal proliferation. Immunostaining for phosphohistone H3 (p-H3) as a mitotic marker in WT and Lrrc10−/− heart sections showed no alterations in proliferation at E13.5 or one month of age. (PDF) [file pone.0051621.s001.pdf]

## Supplemental Figure 2.

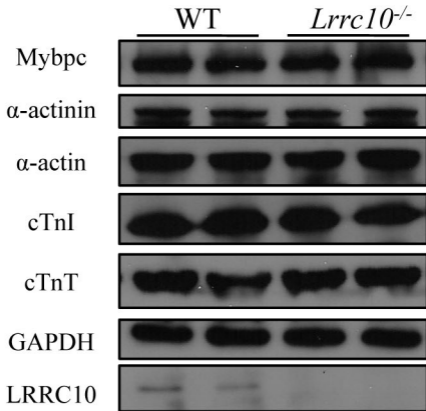

Supplement: Figure S2 — Myofibril protein expression in Lrrc10−/− hearts. No significant difference in protein expression of myofibril proteins between WT and Lrrc10−/− hearts at four months of age was detected by Western blotting (n = 3–4) using various antibodies as indicated. cTnI, cardiac troponin I; cTnT, cardiac troponin T; Mybpc, myosin binding protein-C. (PDF) [file pone.0051621.s002.pdf]
