# Supplementary material for: Ablation of the Cardiac-Specific Gene Leucine-Rich Repeat Containing 10 (Lrrc10) Results in Dilated Cardiomyopathy
Source: PLoS One. 2012 Dec 7;7(12):e51621. doi: 10.1371/journal.pone.0051621 (PMC3517560; doi:10.1371/journal.pone.0051621)
Supplement: Materials and Methods S1 — Supplemental Methods. (DOCX) [file pone.0051621.s003.docx]

**Supplemental Methods**

*Microarray Analysis*

Microarray analyses were performed using RNA isolated from wildtype or *Lrrc10^-/-^* mouse hearts at two month of age. Standard clustering of microarray data obtained from three independent biological replicates was performed using EDGE3 [1] to obtain a data set of probes with a p-value<0.05 and minimum processed signal of 20, which was further sorted into probes with consistently increased or decreased expression at or above 1.2-fold change in all three chips. Dysregulated pathways were determined by querying data sets of upregulated or downregulated genes in DAVID [2,3] after background normalization to the original list of probes (p<0.01, processed signal ≥50) obtained by standard clustering of data from all three chips. Significantly dysregulated pathways were determined using the functional analysis tool in DAVID to identify KEGG pathways that were enriched in consistently up or downregulated genes.

*Quantitative Real Time PCR*

qRT-PCR was performed using FastStart SYBR Green Master (Roche) on a BioRad iCycler. Data were generated using the standard curve method and normalized to 18S expression. All samples were assayed in duplicate with nearly identical replicate values. Primer sequences for ND1, cytb, Cox5a, and ATP6 were previously published [4]. All primers were thoroughly evaluated by melt curve analysis to ensure the amplification of a single, desired amplicon. The following primers were designed using Primer Express 1.0 (ABI Prism) and used for qRT-PCR experiments:

18S for 5’CGCCGCTAGAGGTGAAATTCT3’

18S rev 5’CGAACCTCCGACTTTCGTTCT3’

ANF (*Nppa*) for 5’GTGGACTAGGCTGCAACAGCTT3’

ANF (*Nppa)* rev 5’ACACACCACAAGGGCTTAGGA3’

β-MHC (*Myh7*) for 5’CAGCCATGCCAACCGTATG3’

β-MHC (*Myh7*) rev 5’TTCCACGATGGCGATGTTC3’

BNP for (*Nppb*) 5’TTCACCAATGGTGACAAAATCG3’

BNP rev (*Nppb*) 5’CAAGCTTTGGTGCCGTTGA3’

Cytc for 5’CAGGAGGCAACTGTCTATTCTTGAC3’

Cytc rev 5’TTGCAGATCAACTATAAAGAAGTGTTA3’

Cox7a1 for 5’GACTGACCATGACGCTGACTCT3’

Cox7a1 rev 5’TTAGCATATTTATTGATGTTTGTCCAA3’

Atp5c1 for 5’GTCATCACAAAGGAGTTGATTGAAAT3’

Atp5c1 rev 5’TGGACCAAGTTTCTTCTGACAAAG3’

Atp5o for 5’GTGCACAGTGACCACAGCATCT3’

Atp5o rev 5’TAACTCAGAGAGAACAGCGTCATCTA3’

Atp5h for 5’TGGCTTGGTGGATGATTTTGA3’

Atp5h rev 5’CAAACTCAGCACAGCTCTTCACAT3’

Ndufb9 for 5’TCCAAGAGAGAGCAGTGGAAGAA3’

Ndufb9 rev 5’AGGAGGCAAAGCTTCAGTCATAA3’

Ndufa13 for 5’TCGAAGGTGAAGCAGGACATG3’

Ndufa13 rev 5’CTCATCATTCTCCAGTAGCCAAAG3’

Slc25a4 for 5’TGACACTGTTCGTCGTAGGATGA3’

Slc25a4 rev 5’CAATCTTCCTCCAGCAGTCAAGT3’

Tnni3 for 5’CAAAAGTCACCAAGAACATCACTG3’

Tnni3 rev 5’TGCCACGGAGGTCATAGATCT3’

Tnnt1 for 5’AGAAATATGAGATCAATGTGCTCTACAAC3’

Tnnt1 rev 5’GTCCTGGCAGTCTCACTTCCA3’

Tpm1 for 5’GACCACGCTCTCAACGATATG3’

Tpm1 rev 5’GACATCCAGCTTGACGAAGGA3’

Mybpc3 for 5’CCTGGAGGTGCGAGTTCCT3’

Mybpc3 rev 5’GCATCCATTTGTACCTAGCCATCT3’

Mybpc2 for 5’TTTGATGCAGGGACCTATTCCT3’

Mybpc2 rev 5’GTTCTGATGACTTATTTAGCAGGTGATG3’

**REFERENCES**

1. Vollrath AL, Smith AA, Craven M, Bradfield CA (2009) EDGE(3): a web-based solution for management and analysis of Agilent two color microarray experiments. BMC Bioinformatics 10: 280.

2. Sherman BT, Huang da W, Tan Q, Guo Y, Bour S, et al. (2007) DAVID Knowledgebase: a gene-centered database integrating heterogeneous gene annotation resources to facilitate high-throughput gene functional analysis. BMC Bioinformatics 8: 426.

3. Huang da W, Sherman BT, Tan Q, Kir J, Liu D, et al. (2007) DAVID Bioinformatics Resources: expanded annotation database and novel algorithms to better extract biology from large gene lists. Nucleic Acids Res 35: W169-175.

4. van den Bosch BJ, van den Burg CM, Schoonderwoerd K, Lindsey PJ, Scholte HR, et al. (2005) Regional absence of mitochondria causing energy depletion in the myocardium of muscle LIM protein knockout mice. Cardiovasc Res 65: 411-418.
